# Advances in glycerol metabolism comprehension in yeasts: from regulation to metabolic engineering strategies

**DOI:** 10.1007/s11274-026-05230-3

**Published:** 2026-08-29

**Authors:** Juliana Silva Carneiro Fonseca, Wendel Batista da Silveira

**Affiliations:** 1 Department of Microbiology, Universidade Federal de Viçosa, Viçosa, 36570-900 MG Brazil; 2 Institute of Biotechnology Applied to Agriculture (BIOAGRO), Universidade Federal de Viçosa, Viçosa, 36570-900 MG Brazil

**Keywords:** Glycerol, Non-conventional yeasts, *Yarrowia lipolytica*, Metabolic pathways, Biotechnological innovation

## Abstract

Glycerol is a polyol that can be produced either chemically from oils or propylene, or biologically by yeasts, mainly under osmotic stress. Currently, glycerol is an abundant byproduct generated during biodiesel manufacturing that can be used as substrate in fermentative processes. Its efficient assimilation varies widely among yeast species; therefore, understanding the regulation of both transport and catabolism is pivotal for optimizing biotechnological processes based on this carbon source. This review addresses current knowledge on the regulatory networks controlling glycerol metabolism in yeasts, compassing transport mechanisms, metabolic pathways, transcriptional control and enzyme regulation. We highlight the distinct roles of the Stl1p symporter and Fps1p aquaglyceroporin in mediating glycerol flux across the plasma membrane, as well as the species-specific reliance on either the glycerol-3-phosphate (G3P) or dihydroxyacetone (DHA) pathway for glycerol assimilation. Classical biochemical studies have shown that glycerol catabolic enzymes are tightly regulated by carbon source availability, osmotic conditions, and feedback inhibition. Recently, transcriptomic and genetic analyses, particularly in *Yarrowia lipolytica*, show that the glycerol metabolism is governed by complex interactions between catabolite repression, nutrient signaling, and metabolic intermediates such as G3P, which acts as a key regulatory signal. Despite significant advances, regulatory mechanisms remain elusive in most non-conventional yeasts, underscoring the need for broader comparative studies. In addition, we review major metabolic engineering strategies aimed at enhancing glycerol utilization or redirecting carbon flux toward targeted bioproducts, emphasizing how mechanistic insights into glycerol metabolism and its regulation can guide the development of engineered strains with improved features for industrial applications. Lastly, we pointed out promising avenues for future research and biotechnological innovation.

**Graphic abstract:**

**Figure:**
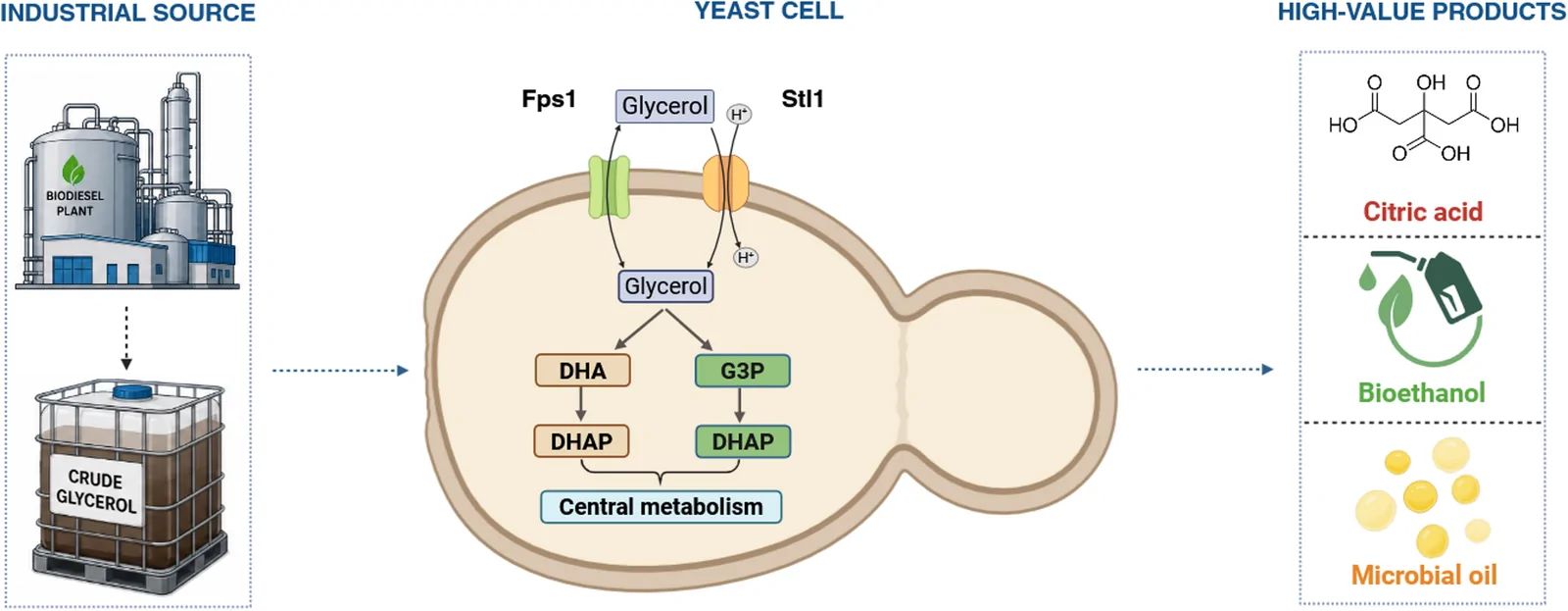

## Introduction

Glycerol is a polyol that can be produced chemically from oils or propylene, or biologically through microbial synthesis at high concentrations by certain osmophilic yeasts (Semkiv et al. 2017b). In recent years, glycerol has attracted considerable attention as a carbon source for the development of fermentation media in bioprocesses, as it has been abundantly generated as a byproduct of biodiesel manufacturing (Swinnen et al. 2013). This polyol can be assimilated by some microorganisms and converted into bioproducts and value-added metabolites. Glycerol has already been used as carbon source for citric acid production by the oleaginous yeast *Yarrowia lipolytica* (Rzechonek et al. 2019), ethanol by an engineered strain of *Saccharomyces cerevisiae* (Yu et al. 2010; Khattab and Watanabe 2021), and microbial oil by *Cryptococcus curvatus* (Liang et al. 2010), *Rhodotorula glutinis* (Sineli et al. 2022) and *Rhodotorula toruloides* (Faife et al. 2025).

The large surplus of crude glycerol generated by the biodiesel industry has spurred extensive research into microbial platforms capable of converting this low-cost substrate into high-value chemicals and fuels, thereby improving the economic viability of biodiesel production (Xiberras et al. 2019). However, the efficiency of glycerol utilization varies markedly across yeast species, presenting both a challenge and an opportunity for metabolic engineering (Klein et al. 2017). While *Y. lipolytica* readily metabolizes glycerol and displays high growth rates, the industrial workhorse *S.cerevisiae* is generally a poor glycerol consumer, which limits its direct application in glycerol-based bioprocesses (Xiberras et al. 2019).

The molecular mechanisms underlying glycerol metabolism in yeasts involve coordinated regulation of transport systems and catabolic and anabolic routes, allowing cells to adjust glycerol flux to their metabolic needs. Glycerol plays a dual role: it serves as a carbon and energy source for growth and, in *S. cerevisiae*, acts as the major osmoprotectant during hyperosmotic shock (Nevoigt and Stahl 1997). Environmental cues, particularly osmotic pressure and the availability of fermentable sugars, determine whether glycerol is synthesized or consumed. Under hyperosmotic stress, the High Osmolarity Glycerol (HOG) pathway triggers rapid glycerol accumulation to restore turgor pressure (Blomberg 2022), while growth on glycerol as the sole carbon source induces either the G3P or the DHA catabolic pathways depending on the species, often under the control of catabolite repression (Sprague and Cronan 1977). A detailed understanding of the regulatory elements governing glycerol transport, assimilation, and osmotic responses is essential for engineering yeast strains with enhanced glycerol-conversion capabilities. This review summarizes the current knowledge on the regulation of glycerol metabolism across yeast species, highlighting the moleculares players and regulatory networks that dictate the metabolic fate of this versatile substrate. We also highlight main perspectives regarding the regulation of glycerol metabolism, underscoring its importance to guide the development of engineered strains with improved features for industrial applications. Lastly, we outline promising avenues for future research and biotechnological innovation.

Even though the foundational pathways of *S. cerevisiae* glycerol metabolism and transport are well-documented, to the best of our knowledge, there are no reviews focusing on providing a comparison between this yeast and non-conventional yeast with potential application in glycerol based-biorefinery. This article distinguishes itself by offering: (i) an integrated thermodynamic comparison of the G3P and DHA catabolic pathways; (ii) a critical analysis of the physiological effects of crude versus pure glycerol on yeast fitness, which has direct industrial implications; (iii) a detailed molecular description of the newly uncovered transcriptional regulatory loops in non-conventional hosts such as *Y. lipolytica*; and (iv) a systematic compile of state-of-the-art quantitative metabolic engineering metrics (including titers, yields, and volumetric productivities) that serves as a guide for selecting and designing strains for the circular bioeconomy.

To ensure a systematic, comprehensive, and reproducible coverage of the literature from 1968 to 2025, relevant peer-reviewed original research and review articles were retrieved from PubMed, Scopus, and Google Scholar using targeted keywords for glycerol transport, catabolism, regulation, and metabolic engineering in yeasts. The search queries combined keywords and Boolean operators as follows: (‘glycerol’ OR ‘polyol’) AND (‘yeast’ OR ‘fungi’ OR ‘Saccharomyces’ OR ‘Yarrowia’ OR ‘Pichia’) AND (‘transport’ OR ‘symport’ OR ‘aquaglyceroporin’ OR ‘catabolism’ OR ‘regulation’ OR ‘metabolic engineering’). Articles were screened based on predefined inclusion and exclusion criteria. Inclusion criteria encompassed: (i) peer-reviewed original research papers and reviews focusing on transport, enzymatic, transcriptional, or metabolic engineering aspects of glycerol in yeasts; (ii) papers providing quantitative bioprocess data (titers, yields, and productivities); and (iii) English-language publications. Exclusion criteria included: (i) non-yeast microbial platforms; (ii) studies focusing solely on industrial biodiesel chemistry without biological bioconversion; and (iii) preprints or gray literature lacking formal peer-review.

## Glycerol metabolism in yeast

### Transport of glycerol

Initially, glycerol is transported into the cell by transporters and protein channels. Conversely, excess intracellular glycerol – such as the generated for NAD^+^ regeneration - is exported to maintain cellular homeostasis. The molecular mechanisms governing glycerol movement across the plasma membrane have been most extensively characterized in *Saccharomyces cerevisiae*. For instance, only a few studies have been conducted in non-*Saccharomyces* yeasts. In *S. cerevisiae*, elucidating the pathways responsible for glycerol transport proved challenging, as multiple possibilities – including passive systems such as simple diffusion and facilitated diffusion, as well as active transport – were initially proposed and investigated. Herein, we will summarize the current understanding of glycerol transport in yeast, emphasizing the key moleculares players involved and their physiological relevance.

Glycerol transport in yeast can be mediated by the Stl1p transporter (*Sugar Transporter-Like 1*) or the Fps1p protein channel. The Stl1p transporter, which mediates active glycerol transport via proton symport (Ferreira et al. 2005), is part of the MFS (*Major Facilitator Superfamily*), the largest family of transporters (Pao et al. 1998). On the other hand, the Fps1p is classified as an aquaglyceroporin from the family MIP (*Major intrinsic proteins*) (Luyten et al. 1995) and shows high sequence similarity to the glycerol facilitator GlpF from *Escherichia coli*, the only transporter responsible for glycerol uptake in that bacterium (Heller et al. 1980). This protein channel facilitates the movement of small, uncharged molecules like water and glycerol via facilitated diffusion. In addition to glycerol uptake, the Fps1p channel also plays a critical role in osmoregulation, whose key function is the rapid efflux of intracellular glycerol following a sudden shift from high to low external osmolarity, a process essential for regulating turgor pressure and preventing cell rupture (Klein et al. 2016, 2017).

Importantly, the *S. cerevisiae* growth rate in culture media containing glycerol is low. Furthermore, many *S. cerevisiae* strains do not grow well in minimal media containing only glycerol as the sole carbon and energy source, requiring culture medium supplementation (Swinnen et al. 2013). Ferreira et al. (2005) revealed the crucial function of Stl1 in *S. cerevisiae* by the deletion of the *STL1* gene, which abolished active glycerol transport, in turn growth. In other yeasts as *Candida albicans* (Kayingo et al. 2009), *Komagataella phaffii* (Zhan et al. 2016), *Wickerhamomyces anomalus* (Cunha et al. 2019) and *Kluyveromyces marxianus* (Zhang et al. 2020), the glycerol uptake takes place through a glycerol/H^+^ symport mechanism. In *S. cerevisiae*, several early experimental studies demonstrated a saturable glycerol uptake component, which was initially believed to be connected with the Fps1 protein (Luyten et al. 1995; Sutherland et al. 1997). However, subsequent studies clarified that Fps1p mainly facilitates glycerol efflux for osmotic regulation, whereas active glycerol uptake is mediated exclusively by the Stl1p transporter (Tamás et al. 1999; Ferreira et al. 2005). The initial observation of saturable glycerol uptake kinetics in *S. cerevisiae* was shown to be an artifact caused by significant Gut1 activity in glucose-grown cells or cells harvested during diauxic shift (Oliveira et al. 2003). Deletion of the *GUT1* gene abolished this effect, demonstrating that Gut1 interfered with the experimental setup. These findings are consistent with the observation of Luyten et al. (1995), that is, the growth of *S. cerevisiae* on glycerol as the sole carbon source was unaffected in *fps1*Δ mutants. Indeed, further studies confirmed that the primary function of Fps1 in *S. cerevisiae* is to control glycerol export during osmoregulation rather than glycerol uptake (Tamás et al. 1999; Oliveira et al. 2003). In this sense, Oliveira et al. (2003) pointed out that Fps1 should not be considered a glycerol facilitator in the context of glycerol uptake, as it does not exhibit Vmax values consistent with facilitated diffusion-type transport.

Before Stl1 has been demonstrated as the main mediator of glycerol uptake in *S. cerevisiae*, attention was given on the gene *GUP1* and its paralog *GUP2*, which were suggested to encode proteins responsible for glycerol active transport (Holst et al. 2000). *GUP1* and *GUP2* encode membrane proteins with multiple predicted transmembrane domains. Holst et al. (2000) showed that the Gup1 protein is critical not only for robust growth in media where glycerol is the sole carbon source, but also for mitigating osmotic stress-induced cell death in *gpd1*Δ *gpd2*Δ mutants. Indeed, deletion of *GUP1* in a *gpd1*Δ *gpd2*Δ mutant diminishes the protective effect of glycerol supplementation under hyperosmotic conditions, indicating that Gup1 also contributes to glycerol uptake. However, the theory that *GUP1* and *GUP2* encode glycerol transporters in *S. cerevisiae* was later undermined. Even prior to the identification of the *STL1* encoded glycerol/H^+^ symporter, comparative studies had shown a key inconsistency: glycerol uptake activity is transiently enhanced upon switching to media containing glycerol or high salt concentrations, whereas the expression patterns of *GUP1* and *GUP2* were found to be constitutive, suggesting they do not mediate the regulated uptake process (Oliveria and Lucas, 2004). Moreover, double deletion mutant *gup1*Δ *gup2*Δ still show active glycerol uptake by glycerol/H^+^ symport under salt stress (Neves et al. 2004), showing that these genes might affect glycerol uptake, but in an indirect way.

Unlike *S. cerevisiae*, the yeast *Yarrowia lipolytica* exhibits high specific growth rates using glycerol as the sole carbon source; furthermore, it co-assimilates glycerol and glucose when both sources are available (Liu et al. 2013). Erian et al. (2022) demonstrated that *Y. lipolytica* possesses 10 putative glycerol transporters. When six of them were expressed in an *S. cerevisiae* strain lacking glycerol transport (*stl1*Δ), the glycerol uptake was restored. Of the six transporters, four are similar to Stl1p and two are similar to Fps1p.

Similar to *Y. lipolytica*, the yeasts *Cyberlindnera jadinii* and *Pachysolen tannophilus* are capable of transporting glycerol through the Fps1p channel, which has approximately 400 amino acid residues. In yeasts such as *S. cerevisiae*, *K. marxianus*, *Kluyveromyces lactis*, and *Zygosaccharomyces rouxii*, the Fps1p channel has approximately 600 amino acids and is not capable of mediating glycerol influx (Klein et al. 2016; Liu et al. 2013). In *S. cerevisiae* (Mollapour and Piper 2007) and *Candida glabrata* (Beese-Sims et al. 2012), this protein channel possesses a long N-terminal domain, which is a target of the MAPK kinase, part of the HOG (High Osmolarity Glycerol) signaling pathway that controls the channel’s opening and closing in response to osmotic stress.

**Fig. 1:**
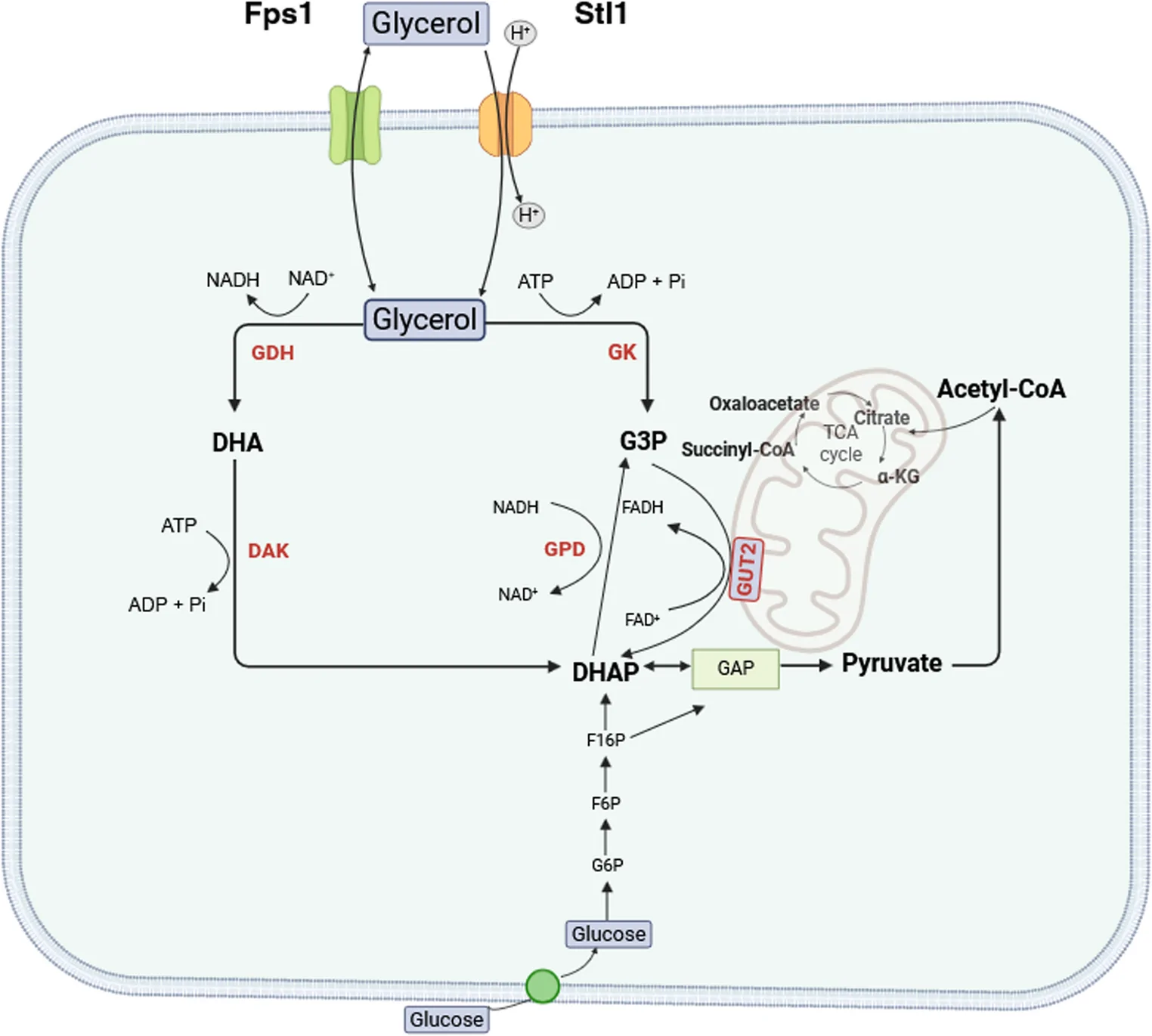
Glycerol uptake and catabolic pathways in yeast. dihydroxyacetone (DHA); glycerol dehydrogenase (GDH); dihydroxyacetone phosphate (DHAP); dihydroxyacetone kinase (DAK); glycerol-3-phosphate (G3P); glycerol kinase (GK); glycerol-3-phosphate dehydrogenase (GUT2); glycerol-3-phosphate dehydrogenase (GPD); glyceraldehyde-3-phosphate (GAP)

### Physiological impacts of pure *versus* crude glycerol in yeast bioprocesses

It is not worthy to distinguish between the utilization of pure glycerol and crude glycerol in yeast bioprocesses. While pure glycerol is a highly consistent and refined carbon source, its high cost restricts its commercial viability for bulk biochemical production. Otherwise, crude glycerol—the primary byproduct of the biodiesel manufacturing industry—is cheap and abundant, representing up to 10% (w/w) of the total biodiesel output (Rzechonek et al. 2019). However, crude glycerol is highly heterogeneous and typically contains only 60% to 80% pure glycerol. The remaining fraction consists of water, inorganic salts (primarily NaCl or KCl, up to 8%), methanol (0.1% to 1%), soaps, heavy metals, and free fatty acids (FFAs) (Jagtap et al. 2021), which impair the yeast growth. The high concentration of sodium or potassium salts can trigger severe osmotic stress and ionic toxicity, leading to intracellular dehydration and metabolic arrest. Methanol, even at low residual concentrations, is toxic to most non-methylotrophic yeasts due to the accumulation of formaldehyde and formate during non-specific oxidation. Soaps can disrupt plasma membrane integrity, permeabilizing the cell and causing the leakage of vital intracellular components. The tolerance to these compounds is species-dependent. *S*. *cerevisiae* is sensitive to the salt and methanol impurities in crude glycerol, exhibiting a prolonged lag phase and reduced growth rates (Xiberras et al. 2019). In contrast, some non-conventional yeasts exhibit higher tolerance than *S. cerevisiae*. *Y. lipolytica* is naturally halotolerant and can metabolize the fatty acid impurities via its extensive mitochondrial beta-oxidation pathway, converting these contaminants into additional carbon feeds (Yuzbasheva et al., 2018). *Debaryomyces hansenii*, which is also a halotolerant yeast, relies on its unique sodium-pump-driven osmoregulatory machinery to thrive in crude glycerol containing high salt loads, maintaining intracellular homeostasis without compromising metabolic productivity (Adler et al. 1985; Navarrete et al. 2021). Therefore, the selection of yeast to be applied in glycerol-based biorefinery must account for these chemical impurities, as the use of crude glycerol is pivotal in terms of economic feasibility.

### Glycerol catabolism

Glycerol is assimilated by yeasts through two distinct metabolic routes, both converging on the synthesis of the central intermediate dihydroxyacetone phosphate (DHAP), which feeds into glycolysis or gluconeogenesis (Semkiv et al. 2017a, b) (Fig. 1).

The first route is the Glycerol-3-Phosphate (G3P) catabolic pathway. This pathway begins with the phosphorylation of glycerol to L-glycerol-3-phosphate (G3P), catalyzed by glycerol kinase (GK/GUT1). G3P is then oxidized to DHAP by the FAD-dependent mitochondrial glycerol-3-phosphate dehydrogenase (mG3PDH/GUT2) (Klein et al. 2017). This dehydrogenase is characteristically located on the outer surface of the inner mitochondrial membrane, channeling electrons from FADH_2_ directly into the respiratory chain. The second route is the Dihydroxyacetone (DHA) catabolic pathway. This pathway starts with the oxidation of glycerol to dihydroxyacetone (DHA), a reaction catalyzed by an NAD^+^-dependent glycerol dehydrogenase (GDH). DHA is subsequently phosphorylated by DHA kinase (DAK) to form DHAP (da Silva et al. 2009; Klein et al. 2017).

In *S. cerevisiae*, glycerol catabolism has been extensively studied. Sprague and Cronan (1977) demonstrated that, although *S. cerevisiae* possesses genes encoding all four enzymes involved in both catabolic pathways, glycerol assimilation occurs exclusively via the G3P pathway. Deletion of the *GUT1* or *GUT2* genes — which encode glycerol kinase (GUT1) and mitochondrial glycerol-3-phosphate dehydrogenase (GUT2), respectively — abolishes the yeast’s ability to utilize glycerol. This finding illustrates that, although a yeast may carry homologous genes for enzymes in multiple pathways, only a single pathway can be responsible for effective glycerol assimilation.

In *K. phaffii*, glycerol is mainly metabolized through the G3P pathway. Tomàs-Gamisans et al. (2019) showed by using ¹³C metabolic flux analysis, that *K. phaffii* primarily channels glycerol carbon through glycolysis and the tricarboxylic acid (TCA) cycle, with only a small contribution from the glyoxylate shunt. This metabolic preference minimizes the use of the carbon-conserving glyoxylate pathway and instead promotes complete carbon oxidation via the TCA cycle, thereby enhancing the activity of NADPH-generating enzymes, such as NADP⁺-dependent isocitrate dehydrogenase. Although the oxidative pentose phosphate pathway remains weakly active during glycerol assimilation, auxiliary reactions in the TCA cycle supply sufficient NADPH to support biosynthetic processes and secondary metabolite formation.

In contrast to *S. cerevisiae* and *K. phaffii*, the yeast *Schizosaccharomyces pombe* assimilates glycerol only through DHA pathway (Matsuzawa et al. 2010). The strain used in this study was unable to grow in media containing glycerol as the sole carbon source. Glycerol assimilation only occurred in the presence of other carbon sources, such as galactose and ethanol. Nevertheless, the authors identified the gene *gld1*^+^ whose product is homologous to bacterial glycerol dehydrogenases (GDH). Its deletion caused a reduction in NAD^+^ - dependent GDH activity and prevented glycerol assimilation.

In *Kluyveromyces marxianus*, glycerol catabolism has been recently elucidated through systematic gene disruption using a transient CRISPR/Cas9 system, revealing that glycerol is predominantly metabolized via G3P pathway, while the DHA route functions as an auxiliary pathway (Ren et al. 2022). Deletion of *GUT1* or *GUT2* markedly impaired growth on glycerol, confirming this pathway as the main route for glycerol catabolism. The DHA pathway plays a secondary but physiologically relevant role, since *DAK1* deletion reduced glycerol utilization and increased sensitivity to exogenous DHA. Mutants lacking key genes from both pathways were unable to grow on glycerol in minimal medium, indicating that these routes together account for glycerol assimilation. Additionally, high glycerol concentrations partially restored growth and enhanced enzyme activities, suggesting compensatory metabolic responses under substrate-rich conditions (Ren et al. 2022).

In *K. lactis*, glycerol catabolism has been shown to rely primarily on the G3P pathway, similarly to other respiratory yeasts, such as *K. phaffii* (Tomàs-Gamisans et al. 2019) and *K. marxianus* (Ren et al. 2022), even though regulatory and physiological features distinguish this species. Functional analyses demonstrated that genes homologous to *GUT*1 and *GUT*2 are essential for growth on glycerol as the sole carbon source, confirming that glycerol kinase and mitochondrial glycerol-3-phosphate dehydrogenase constitute the main catabolic route. Disruption of these genes severely impaired glycerol utilization, whereas genes encoding enzymes of the DHA pathway were not sufficient to sustain growth independently, indicating that this route does not play a major assimilatory role under standard conditions. Moreover, glycerol metabolism in *K. lactis* is tightly linked to respiratory activity and mitochondrial function, consistent with its predominantly respiratory lifestyle and limited fermentative capacity. These findings reinforce the concept that, although both assimilatory pathways may be genetically present, the G3P route is functionally dominant in *K. lactis* (Saliola et al. 2008).

The selection of either the G3P or the DHA pathway as the primary route for glycerol assimilation carries severe thermodynamic and bioenergetic consequences, particularly under varying oxygen availability. The G3P pathway is compartmentalized, requiring the cytoplasmic conversion of glycerol to G3P by GK, followed by its transport and oxidation on the outer surface of the inner mitochondrial membrane by mG3PDH (Gut2p) (Klein et al. 2017). This second step is coupled directly to the mitochondrial respiratory chain via FAD reduction. From a thermodynamic standpoint, the oxidation of G3P to DHAP is highly exergonic when coupled to oxygen as the terminal electron acceptor, as the electrons bypass the cytoplasmic NADH pool and enter the electron transport chain at the level of coenzyme Q (generating ~ 1.5 ATP per FADH_2_ oxidized). However, this pathway is strictly dependent on active cellular respiration. Under anaerobic or oxygen-limited conditions, the absence of oxygen blocks the reoxidation of mitochondrial FAD, completely halting the G3P pathway. This explains why *S. cerevisiae* cannot utilize glycerol anaerobically, even when active transporters are overexpressed (Sprague and Cronan 1977; Geertman et al. 2006).

In contrast, the DHA pathway is strictly cytosolic and operates independently of direct mitochondrial respiration (Xiberras et al. 2020). Glycerol is oxidized to DHA by a cytosolic NAD^+^-dependent GDH, generating cytosolic NADH, which is subsequently phosphorylated to DHAP by DAK at the expense of one ATP molecule (Liu et al. 2024; Sun et al. 2021). Although the initial oxidation step is thermodynamically less favorable (ΔG°’ = +25 kJ/mol) and requires a high NAD^+^/NADH ratio to proceed, it yields cytosolic NADH directly (Sun et al. 2021). This cytosolic NADH represents vital reducing equivalents that can be directly routed to biosyntheses or used to balance redox states during fermentation under oxygen-limited conditions. However, this direct generation of cytosolic NADH requires tight redox balancing, as respiratory mechanisms such as external NADH dehydrogenases compete for the same reducing pool (Aßkamp et al. 2019). If the cell cannot reoxidize this NADH pool through active NADH-dependent pathways like ethanol or 1,2-propanediol synthesis, the resulting redox imbalance restricts growth and stalls glycerol assimilation (Aßkamp et al. 2019; Islam et al. 2017). Thus, while the G3P pathway is more energetically efficient in fully aerobic cultures, the DHA pathway offers greater metabolic flexibility in redox-balanced anaerobic or microaerophilic processes, making it a critical target for industrial metabolic engineering.

## Regulation of glycerol metabolism

### Transcriptional regulation

The transcriptional regulation of glycerol metabolism in yeasts is influenced by the available carbon source, reflecting a complex interplay between catabolite repression, induction, and nutrient signaling.

**Fig. 2:**
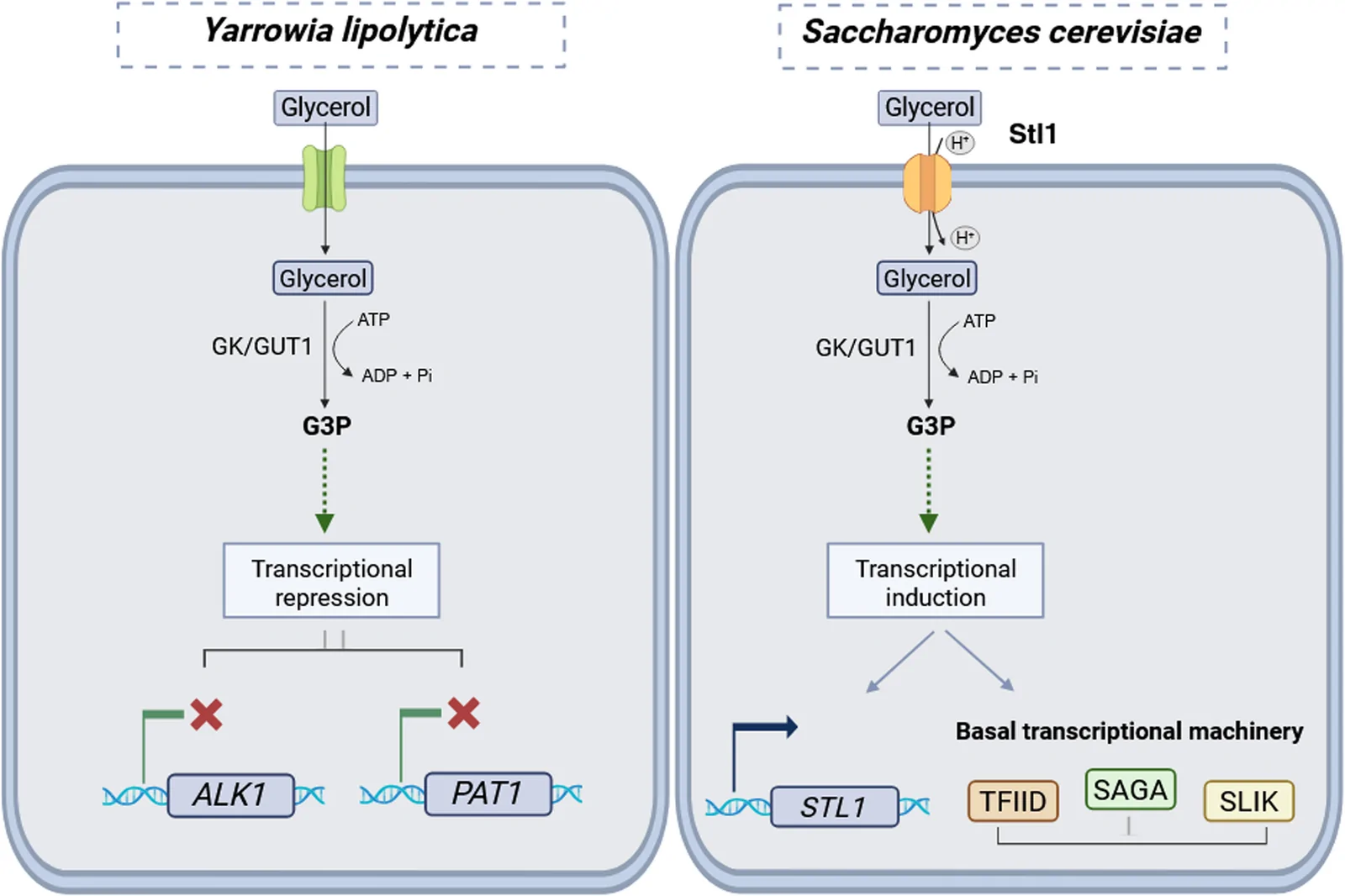
Comparative mechanisms of glycerol-induced transcriptional regulation in *Yarrowia lipolytica* and *Saccharomyces cerevisiae*

In *Y. lipolytica*, this regulatory response is dependent on the initial step of glycerol assimilation, demonstrating that a metabolic intermediate acts as the core regulatory signal (Mori et al., 2013). Mutational analysis, specifically the deletion of the glycerol kinase gene (*GUT*1), revealed that glycerol must be converted to glycerol-3-phosphate (G3P) by Gut1p to trigger downstream transcriptional repression (Fig. 2). In deletion mutants lacking glycerol kinase (Δ*gut1*), the glycerol-mediated repression of target genes such as *ALK1* and *PAT1* was abolished. In contrast, deletion of the mitochondrial glycerol-3-phosphate dehydrogenase gene (Δ*gut2*) did not relieve this repression. This result suggests that G3P, the product of Gut1p activity, is the essential intracellular effector that signals the availability of glycerol and initiates the process of transcriptional repression on glycerol-responsive genes (Mori et al., 2013).

In the budding yeast *S. cerevisiae*, the adaptation from glucose-based fermentative growth to glycerol-based respiratory growth involves a comprehensive metabolic and structural remodeling of the cell. Indeed, transcriptome profiling during this adaptation revealed that the transcriptional changes extend beyond a simple switch of metabolic pathways, eliciting an extensive adaptation that includes the up-regulation of genes associated with the oxidative and osmotic stress responses (Roberts and Hudson 2006). A critical component of this regulation is the strong induction of key glycerol metabolism genes, such as the glycerol/H^+^ symporter gene *STL*1, which was induced hundreds of times over basal levels during continuous glycerol growth. Furthermore, the switch to glycerol as the sole carbon source resulted in widespread differential expression of genes specifying transcription factors, affecting over 200 transcription-related genes in continuously glycerol-grown cells. Importantly, this adaptation also led to an ordered series of transcriptional changes in components of the basal transcriptional machinery, specifically for genes encoding subunits of the TFIID, SAGA, and SLIK complexes, as well as all three RNA polymerases, suggesting a modulation of the fundamental transcription apparatus during adaptation to respiratory growth (Roberts and Hudson 2006) (Fig. 2).

Unlike non-conventional respiratory yeasts, *S. cerevisiae* is a Crabtree-positive yeast that prefers fermentative metabolism over respiration when glucose is abundant, even in the presence of oxygen (Malina et al. 2021). Under these conditions, a highly coordinated and tight process of glucose (carbon catabolite) repression blocks the transcription of genes encoding proteins required for the utilization of alternative carbon sources, such as glycerol (Carlson 1999; Turcotte et al., 2009). This glucose repression is the primary cause for the relatively poor use of glycerol by *S. cerevisiae* (Xiberras et al. 2019). The repression is mainly mediated by the DNA-binding transcriptional repressor Mig1, which recruits the Ssn6-Tup1 co-repressor complex to the promoters of target genes—including *STL1* and *GUT1*—under glucose-replete conditions, thereby blocking glycerol transport and the initial step of its catabolism (Kayikci and Nielsen 2015).

The relief of this glucose repression upon glucose exhaustion relies heavily on the SNF1 (sucrose non-fermenting 1) protein kinase pathway, the yeast ortholog of mammalian AMP-activated protein kinase (AMPK) (Carlson 1999). When glucose levels drop, the Snf1 kinase complex is activated by phosphorylation. Activated Snf1 directly phosphorylates the repressor Mig1, promoting its export from the nucleus to the cytoplasm, thereby relieving the transcriptional repression on alternative carbon source utilization genes (Kayikci and Nielsen 2015). Concurrently, activated Snf1 phosphorylates and activates key zinc-finger transcription factors, such as Cat8 and Sip4, which bind to carbon source-responsive elements (CSRE) in the promoters of *GUT1* and *GUT2*, driving their transcriptional induction and enabling respiratory glycerol catabolism (Schüller 2003; Turcotte et al., 2009).

In contrast, the cAMP-dependent Protein Kinase A (PKA) signaling pathway plays an opposing, repressive role in regulating alternative carbon source use. In the presence of glucose, extracellular glucose sensing via the Gpr1-Gpa2 GPCR system and intracellular glucose phosphorylation trigger cAMP synthesis, which activates PKA (Rolland et al. 2000). High PKA activity promotes rapid fermentative growth and actively represses alternative carbon utilization and respiratory genes (including *STL1* and *GUT1*) while inactivating the general stress-responsive transcription factor Msn2, which are otherwise required for full environmental adaptation (Kayikci and Nielsen 2015). Consequently, the tight interplay between the SNF1 pathway (promoting derepression) and the PKA pathway (maintaining glucose-induced repression and fermentation) represents the central molecular mechanism dictating *S. cerevisiae* physiological response to carbon source availability. The dominant glucose repression under standard conditions remains a major molecular barrier for the efficient biotechnological utilization of glycerol in this industrial host (Kayikci and Nielsen 2015; Xiberras et al. 2019).

While studies in *S. cerevisiae* have covered general glucose repression, osmotic regulation, and redox-related responses, comprehensive analyses directly addressing glycerol-specific transcriptional control are still limited. Current knowledge regarding the role of glycerol or its intermediates as direct transcriptional signals remains overwhelmingly centered on *Y. lipolytica*. This lack of comparative transcriptional studies restricts our ability to discern conserved versus species-specific regulatory mechanisms, underscoring the need for broader, systematic investigations across diverse yeast strains to fully elucidate how glycerol functions as both a metabolic substrate and a transcriptional signal in fungal physiology.

### Regulation of key enzymes of glycerol metabolism

The regulation of glycerol catabolism in yeasts encompasses the coordinated control of the key enzymes glycerol kinase (GK), glycerol-3-phosphate dehydrogenase (G3PDH), and glycerophosphatase, which are responsible for governing the balance between glycerol catabolism and its biosynthesis. Previous biochemical studies pointed out that the activities of these enzymes are tightly modulated by both environmental and metabolic signals, including carbon source availability, osmotic stress, and feedback inhibition by glycolytic intermediates.

Glycerol kinase catalyzes the phosphorylation of glycerol to glycerol-3-phosphate (G3P), the entry point of glycerol into central metabolism (Hagopian et al., 2008). The classic work conducted by Gancedo et al. (1968) demonstrated that GK in *Candida utilis* is an inducible enzyme, as its synthesis increases markedly when cells are grown on glycerol compared to glucose. This induction is attributed to the presence of glycerol or its metabolic derivatives as inducers, while glucose exerts catabolite repression on the enzyme. Moreover, *C. utilis* grown on glycerol exhibited up to sevenfold higher GK activity than glucose-grown cells, confirming the strong dependence of enzyme levels on carbon source. The authors also described AMP as a specific inhibitor of GK, conferring physiological significance under anaerobic conditions, preventing futile ATP expenditure when oxidative metabolism is inactive. In contrast, the enzyme showed substrate specificity toward polyols possessing three appropriately oriented hydroxyl groups, such as glycerol and dihydroxyacetone, but not shorter or longer chain analogs, defining a clear stereochemical requirement for substrate binding.

Complementary findings by Sprague and Cronan (1977) in *S. cerevisiae* further elucidated the influence of carbon catabolite repression on the regulation of GK and mitochondrial glycerol-3-phosphate dehydrogenase (mG3PDH, encoded by *GUT2*). In glucose-grown cells, both enzymes exhibited very low activity, whereas growth on non-fermentable carbon sources such as ethanol, lactate, or glycerol markedly increased their levels. The authors demonstrated that the decline in enzyme activity after glucose addition to glycerol-grown cultures was not due to direct enzyme inhibition or degradation, but rather to repression of *de novo* synthesis. Thus, *S. cerevisiae* modulates glycerol catabolic capacity through transcriptional repression of key enzymes under glucose-replete conditions, highlighting a classical model of catabolite repression controlling carbon flux distribution (Sprague and Cronan 1977; Grauslund and Rønnow 2000).

A more detailed analysis of enzyme behavior under stress and respiratory conditions was later provided by Adler et al. (1985) in *Debaryomyces hansenii*, a yeast well-known for its osmotic tolerance (Navarrete et al. 2021). Their results revealed that the NAD-dependent cytosolic G3PDH, which catalyzes the interconversion of dihydroxyacetone phosphate (DHAP) and G3P, displayed the highest specific activity among the enzymes tested and increased approximately twofold under high-salt stress. In contrast, the enzyme’s activity was reduced during the stationary phase or when grown on glycerol as the sole carbon source. Glycerol kinase activity was strongly induced by growth on glycerol, while mG3PDH showed elevated activity both in glycerol-grown and salt-stressed cells. Importantly, glycerol kinase activity was found to be tightly regulated by fructose-1,6-bisphosphate (FBP), a key glycolytic intermediate, which inhibited the enzyme by up to 95%. This inhibition, resembling that observed in *Escherichia coli* GK (Thorner and Paulus 1973), reflects feedback control linking glycolytic flux with glycerol utilization, ensuring metabolic balance between energy production and osmoprotection. The study also demonstrated that removal of low-molecular-weight inhibitors from crude extracts (by gel filtration) led to a 2–10-fold increase in GK activity, reinforcing the idea of metabolite-mediated regulation.

Taken together, these studies delineate a multifaceted regulatory framework in which enzyme induction, catabolite repression, and allosteric inhibition collectively control glycerol metabolism in yeasts. Under gluconeogenic or osmotic stress conditions, glycerol catabolic enzymes are induced to sustain carbon flux and redox balance, while in glucose-rich environments, their synthesis is repressed to favor glycolysis. Moreover, the interplay between intracellular effectors such as FBP and AMP ensures that glycerol metabolism is finely tuned to the cell’s energetic and redox state. This tight regulation allows yeasts to efficiently adjust glycerol flux between catabolic and anabolic routes in response to environmental and metabolic cues (Table 1).

**Table 1:** Summary of key enzymes involved in glycerol metabolism, including their primary functions, regulatory mechanisms, and modulators across different yeast species

Enzyme	Primary Function	Regulatory Mechanism	Signals and Modulators (effect)	Organism	References
Glycerol Kinase (GK)	Phosporylates glycerol to G3P (entry point to metabolism)	Transcriptional Induction	Glycerol: Strong inducer (up to 7-fold activity increase).	*Candida utilis*	Gancedo et al. (1968).
		Catabolite Repression	Glucose: Represses de novo synthesis.	*Saccharomyces cerevisiae*	Sprague and Cronan 1977; Grauslund & Ronnow, 2000.
		Allosteric Inhibition (Feedback)	AMP: Specific inhibitor (prevents futile ATP use).	*Candida utilis*	Gancedo et al. (1968).
			FBP: Strong inhibitor (up to 95%; links glycolytic flux to glycerol use).	*Debaryomyces hansenii*	Adler et al. (1985).
Cytosolic G3PDH (NAD-dependent)	Interconverts DHAP and G3P	Stress Response	High Salt: Increases activity (2-fold) for osmoprotection. Glycerol: Activity reduced when grown on glycerol or during the stationary phase.	*Debaryomyces hansenii*	Adler et al. (1985).
mG3PDH (GUT2) (mitochondrial)	Oxidizes G3P in the respiratory chain	Catabolite Repression	Glucose: very low activity. Non-fermentable sources (ethanol/lactate): Markedly increased levels.	*Saccharomyces cerevisiae*	Sprague and Cronan 1977; Grauslund & Ronnow, 2000.
		Stress Response	High Salt: Elevated activity.	*Debaryomyces hansenii*	Adler et al. (1985).

### Metabolic engineering for glycerol metabolism optimization

Metabolic engineering has been applied to alter the glycerol metabolism with two primary and distinct objectives: optimizing the utilization of crude glycerol as an abundant, inexpensive feedstock for the synthesis of value-added biochemicals, and enhancing the native capacity of yeasts to produce and accumulate glycerol from primary carbon sources like glucose (Table 2).

For bioconversion applications, the oleaginous yeast *Y. lipolytica* is a frequent target due to its inherent lipogenic pathways and natural ability to produce polyols and sugar alcohols such as erythritol, mannitol, and arabitol (Jagtap et al. 2021). Engineering strategies for high-titer erythritol production typically employ a modular approach: initially, degradation and competition modules are blocked (e.g., via *URA*3 counter-selection) to eliminate catabolic pathways and byproduct formation. Subsequently, multi-module gene combinations are expressed to establish a shortened biosynthetic pathway, thereby elevating the synthetic flux toward erythritol. For example, modular engineering of erythritol biosynthesis in *Y. lipolytica* by eliminating degradation pathways (*EYD1*), blocking byproduct synthesis (*ArDH1* and *MDH2*), overexpressing biosynthetic genes (*GUT1*, *ER*, *TKL1*, and *TAL1*), and introducing a heterologous glycerol transporter *ScFPS1* (strain ERY8) - yielded an outstanding concentration of 176.66 g/L of erythritol in fed-batch cultivation. This strategy achieved a conversion yield of 0.631 g/g on glycerol, representing 37.17% improvement compared to the control strain, while eliminating the accumulation of mannitol and arabitol (Huang et al. 2023). Optimization is also achieved by diverting metabolic flow away from glycerol catabolism; for instance, redirecting carbon flux in *Y. lipolytica* by disrupting *GPD1* and *GUT2* led to the hyper-accumulation of free fatty acids (FFAs), reaching a titer of 2.03 g/L in flask-scale, a substantial improvement over the non-engineered control (Yuzbasheva et al., 2018).

Another non-conventional yeast, *K. phaffii*, has been engineered to achieve the synergetic utilization of both glucose and glycerol in order to improve its versatility as a fermentation platform for biochemical production. To overcome glucose-mediated carbon catabolite repression, overexpressing the glycerol catabolic genes *GUT1* and *GUT2* alongside the glycerol transporter GT1 (strain Z2) enabled simultaneous consumption of 18.4 g/L glucose and 10.0 g/L glycerol within 60 h. To further conserve glucose for product synthesis rather than cell growth, flux into glycolisis and the pentose phosphate pathway was restricted. Combining this with the co-expression of the glycerol symporter GT2 (strain Z6) maintained efficient co-utilization while reducing glucose consumption to 9.2 g/L, shortening the lag phase to 18 h. (Wang et al. 2022).

In *S. cerevisiae*, which profits from glycerol’s higher reducing power, a novel approach for producing 1,2-propanediol (1,2-PDO) was developed by implementing the heterologous methylglyoxal (MG) pathway - headlined by methylglyoxal synthase (mgsA) - replacing native FAD-dependent pathway with an NAD^+^-dependent DHA pathway to boost cytosolic NADH, and introducing the glycerol facilitator CjFPS1. To prevent the precursor dihydroxyacetone phosphate (DHAP) from being entirely consumed by the competing glycolytic enzyme triosephosphate isomerase (TPI1), its expression was attenuated. Combining these modules with a second mgsA cassette yielded 2.5 g/L of 1,2-PDO in shake flasks and reached a record titer of 4.3 g/L in controlled bioreactor cultivations (Islam et al. 2017). Host optimization can also be achieved through non-directed methods like Adaptive Laboratory Evolution (ALE), which has been instrumental in identifying genetic targets for improving the intrinsic glycerol assimilation ability of *S. cerevisiae* strains. In this study, serial transfers on glycerol evolved the wild-type strain over 85 generations, increasing its specific growth rate (µ) to 0.14 h^− 1^. Transcriptome analysis guided metabolic engineering strategies, revealing that combining *STL1* overexpression with *RIM15* disruption (*rim15*Δ) significantly reduced the lag phase to approximately 48 h and achieved a specific growth rate of 0.056 h^− 1^ on glycerol. Furthermore, combined overexpression of *STL1* and *HAP4* reduced the lag phase from > 480 h (control) to ~ 150 h (Kawai et al. 2019).

For the second objective, increasing endogenous glycerol production from glucose, particularly under cost-effective anaerobic conditions, the yeast *S. cerevisiae* has been the primary focus of engineering efforts to manipulate the cytosolic redox balance and triose phosphate metabolism. An early strategy involved the inactivation of the *TPI1* gene, which encodes triose phosphate isomerase, thereby forcing the accumulated dihydroxyacetone phosphate (DHAP) to be redirected and reduced by NADH into glycerol. However, complete inactivation of *TPI1* abolishes growth on glucose as a sole carbon source, a defect potentially attributed to the mitochondrial reoxidation of cytosolic NADH, rendering it unavailable for the reduction of DHAP to glycerol (Overkamp et al. 2002). To address this limitation, Overkamp et al. (2002) constructed a quadruple mutant designed to direct cytosolic NADH exclusively toward glycerol formation. Although initial growth was inhibited by glucose repression of essential respiratory enzymes, serial transfers on high-glucose media yielded an adapted strain capable of robust growth (µ = 0.10 h⁻¹). In high-density aerated batch cultures containing 400 g/L glucose, this engineered strain produced more than 200 g/L glycerol, reaching a molar yield close to unity (1.0 mol/mol). To build upon these findings, more advanced strategies for *S. cerevisiae* seek to circumvent the rigid stoichiometric constraints of previous attempts (related to carbon, ATP, or redox) by maintaining flexibility at triosephosphate isomerase and fructose-1,6-bisphosphatase, simultaneously eliminating competing reactions for cytosolic NADH, and enabling oxidative catabolism within the mitochondrial matrix. To accomplish this, Geertman et al. (2006) engineered a strain lacking pyruvate decarboxylases, external NADH dehydrogenases, and the respiratory chain-linked glycerol-3-phosphate dehydrogenase. In aerobic glucose-grown batch cultures, this strain achieved a glycerol yield of 0.90 mol (mol glucose)^−1^. Furthermore, by generating additional cytosolic NADH via formate co-feeding in glucose-limited chemostats, the engineered strain successfully surpassed the conventional stoichiometric ceiling, reaching a record-breaking yield of 1.08 mol (mol glucose)^−1^ the highest glycerol yield reported for *S. cerevisiae* (Geertman et al. 2006). More recently, to maximize this carbon flux without incurring the growth defects of total TPI1 deletion, Semkiv et al. (2017a) combined TPI1 promoter attenuation with the overexpression of a fused *GPD1-GPP2* gene (enhancing glycerol-3-phosphate dehydrogenase and phosphatase activities), an engineered *FPS1* aquaglyceroporin channel, and a truncated *ILV2* gene (acetolactate synthase). This multi-targeted strain achieved a maximum titer exceeding 20 g/L of glycerol from glucose under micro-aerobic conditions and over 16 g/L under strict anaerobic conditions, accompanied by an expected reduction in ethanol and biomass accumulation (Semkiv et al. 2017a).

**Table 2:** Metabolic engineering strategies targeting glycerol metabolism in yeasts

Objective	Target Organism	Engineering Strategy/ Pathway	Result/ Compound Produced	References
I. Optimizing utilization of crude glycerol	*Yarrowia lipolytica*	Modular approach: Block degradation/competition modules (e.g., via URA3 counter-selection); Express multi-module gene combinations for shortened biosynthetic pathway.	Erythritol (High-titer production)	Jagtap et al. 2021; Huang et al. 2023
	*Yarrowia lipolytica*	Deletion of *GPD1* and *GUT2* genes to eliminate glycerol metabolism and produce free fatty acids.	Free Fatty Acids (FFAs)	Yuzbasheva et al., 2018
	*Komagataella phaffii*	Engineered for synergetic utilization of both carbon sources.	Synergetic utilization of Glucose and Glycerol	Wang et al. 2022
	*Saccharomyces cerevisiae*	Implement heterologous Methylglyoxal (MG) pathway from DHAP; Replace native FAD-dependent glycerol pathway; Introduce heterologous glycerol facilitator for uptake.	1,2-Propanediol (1,2-PDO)	Islam et al. 2017
	*Saccharomyces cerevisiae*	Non-directed method: Adaptive Laboratory Evolution (ALE).	Improved Intrinsic Glycerol Assimilation ability.	Kawai et al. 2019
II. Enhancing native capacity to produce glycerol	*Saccharomyces cerevisiae*	Lowering expression of the TPI1 gene (encoding Triose Phosphate Isomerase) to accumulate DHAP.	Increased glycerol yield (by diverting accumulated DHAP toward NADH-dependent reduction).	Semkiv et al. 2017a, b
	*Saccharomyces cerevisiae*	Complete disruption of TPI1 gene conding triose Phosphate Isomerase.	It demonstrated the growth defect on glucose (attributed to the mitochondrial reoxidation of cytosolic NADH, making it unavailable for DHAP reduction).	Overkamp et al. 2002
	*Saccharomyces cerevisiae*	Advanced strategies: Maintain flexibility at triosephosphate isomerase and fructose-1,6-bisphosphatase; Eliminate competing reactions for cytosolic NADH; Enable oxidative catabolism in mitochondria.	Circumvents stoichiometric constraints, improved Glycerol Production.	Geertman et al. 2006

## Conclusions and futures perspectives

This review provided a comprehensive analysis of the transport mechanisms and regulatory networks governing glycerol metabolism in yeasts. By integrating classical biochemical knowledge with the latest findings in genomics and transcriptomics, the distinctive focus of this review lies in its emphasis on the complexity of molecular regulation—specifically transcriptional control and enzymatic regulation—and the functional distinction of glycerol transport systems, particularly in non-conventional yeasts of biotechnological interest. This study detailed the intricate coordination between active transport mediated by the Stl1p symporter and facilitated diffusion via Fps1p aquaglyceroporins, and how the regulation of these transporters is crucial for the metabolic fate of glycerol. We highlighted the species-specific reliance on either the G3P or DHA pathway and, critically, the complex interplay of catabolite repression, nutrient signaling, and metabolic intermediates. This level of regulatory analysis, providing a solid mechanistic basis for rational metabolic engineering, is the central contribution that sets this review apart from existing literature.

Despite significant advancements, particularly in *Yarrowia lipolytica* and *Saccharomyces cerevisiae*, the molecular mechanisms governing glycerol movement across the plasma membrane and its catabolic regulation remain poorly characterized. Future research must prioritize the full characterization of glycerol transport across diverse yeast strains to better understand the conditions and structural features that dictate the function of Fps1p as either an exporter or an importer, and the regulation of Stl1p-like symporters, which are essential for active uptake. Furthermore, the limited nature of current knowledge underscores the need for broader comparative studies. These systematic investigations, involving other promising species, are vital to discern conserved versus species-specific regulatory strategies. Ultimately, the comprehensive mechanistic insights gained from studying these non-conventional yeasts will guide the rational design of engineered strains with improved features for industrial applications. By focusing on optimizing the utilization of crude glycerol, an abundant byproduct of biodiesel manufacturing, these efforts directly contribute to the circular bioeconomy, enhancing the economic viability of bioprocesses through the efficient conversion of a low-cost substrate into high-value chemicals and fuels.

## Data Availability

No datasets were generated or analysed during the current study.
